# Predicted molecular signaling guiding photoreceptor cell migration following transplantation into damaged retina

**DOI:** 10.1038/srep22392

**Published:** 2016-03-03

**Authors:** Uchenna John Unachukwu, Alice Warren, Ze Li, Shawn Mishra, Jing Zhou, Moira Sauane, Hyungsik Lim, Maribel Vazquez, Stephen Redenti

**Affiliations:** 1 Biochemistry Doctoral Program, The Graduate School, City University of New York, New York, NY, USA; 2 Department of Biological Sciences, Lehman College, City University of New York, Bronx, NY, USA; 3 Department of Biomedical Engineering, City College of New York, City University of New York, NY, USA; 4 Neuroscience Doctoral Program, The Graduate School, City University of New York, New York, NY, USA; 5 Departments of Physics and Biology, Hunter College of the City University of New York, New York, NY USA

## Abstract

To replace photoreceptors lost to disease or trauma and restore vision, laboratories around the world are investigating photoreceptor replacement strategies using subretinal transplantation of photoreceptor precursor cells (PPCs) and retinal progenitor cells (RPCs). Significant obstacles to advancement of photoreceptor cell-replacement include low migration rates of transplanted cells into host retina and an absence of data describing chemotactic signaling guiding migration of transplanted cells in the damaged retinal microenvironment. To elucidate chemotactic signaling guiding transplanted cell migration, bioinformatics modeling of PPC transplantation into light-damaged retina was performed. The bioinformatics modeling analyzed whole-genome expression data and matched PPC chemotactic cell-surface receptors to cognate ligands expressed in the light-damaged retinal microenvironment. A library of significantly predicted chemotactic ligand-receptor pairs, as well as downstream signaling networks was generated. PPC and RPC migration in microfluidic ligand gradients were analyzed using a highly predicted ligand-receptor pair, SDF-1α – CXCR4, and both PPCs and RPCs exhibited significant chemotaxis. This work present a systems level model and begins to elucidate molecular mechanisms involved in PPC and RPC migration within the damaged retinal microenvironment.

Photoreceptor loss is a major feature of light-damage and disease induced photoreceptor degeneration, both of which lead to blindness throughout the world^1^,^2^,^3^. There are growing populations at risk of photoreceptor degeneration through age-related macular degeneration and diabetic retinopathy^2^,^4^. Photoreceptor degeneration is often irreversible and there are currently no effective cell replacement therapies. A promising experimental approach under investigation is photoreceptor replacement via sub-retinal transplantation of donor cells^5^,^6^,^7^,^8^,^9^,^10^. Recent cell replacement studies demonstrate feasibility using transplantable postnatal PPCs, culture expanded RPCs, embryonic (ESc)^11^,^12^,^13^,^14^ and induced pluripotent stem cell (iPSc)^15^ derived retina and PPCs. PPCs and RPCs from human ESc and iPSc derived retina remain promising tissue sources for allogeneic and autologous retinal cell transplantation^16^,^17^,^18^.

A major obstacle to photoreceptor replacement remains that ongoing transplantation studies report extremely low levels of transplanted cell morphologic and functional integration^18^,^19^. While variables including age, retinal disease progression, glial scarring and the outer limiting membrane (OLM) integrity can be manipulated to improve migration, many additional factors guiding migration remain to be defined^20^. There is currently limited understanding of the migratory signaling pathways and molecular mechanisms facilitating motility of transplanted PPCs and RPCs in adult, damaged retinal microenvironments. Following transplantation, the path of migration into retina requires PPCs and RPCs to navigate a range of signaling molecules including heparan-chondroitin-proteoglycan moieties of the interphotoreceptor matrix^21^,^22^,^23^ and the OLM comprised of Muller cell processes and apical villi^24^,^25^. In efforts to improve transplantation outcomes, researchers have modified host retina using enhanced growth factor expression^26^,^27^ and disruption of glial scars and outer limiting membrane^20^,^24^,^25^,^28^. These efforts yielded modest improvements in integrating cell numbers and indicate the importance of defining signaling pathways and molecular mechanisms facilitating migration of transplanted cells.

Transplanted PPCs and RPCs have to migrate from within the subretinal space through the interphotoreceptor matrix and into the adjacent outer nuclear layer to integrate with remaining photoreceptors in host retina^19^,^29^,^30^. Host retinal microenvironments present bound and diffusible ligands, which can interact with migratory cell-surface receptors present on transplanted PPCs and RPCs to guide migration^23^,^28^,^31^. In this study, ligands in the extracellular matrix of light damaged neurosensory retina (NSR) and retinal pigment epithelium (RPE) were paired to cognate cell-surface receptors expressed on PPCs using Ingenuity pathway analysis (IPA) of whole genome arrays, simulating migratory interactions present during transplantation. Downstream signaling pathways were modeled and intracellular networks specific for PPC migration were identified with activation state predicted based on gene expression levels. Comparable bioinformatics analyses of retinal gene expression data have been used to predict cell activity in previous studies^32^,^33^,^34^,^35^,^36^,^37^,^38^.

IPA modeling identified several well characterized ligands present in the NSR and RPE that directly interact with PPC migratory receptors including: brain-derived neurotrophic factor (BDNF), stromal-derived factor-1α (SDF-1α), SLIT proteins, insulin-like growth factor (IGF) and glial-derived neurotrophic factor (GDNF). An important migration inducing interaction was the binding of SDF-1α to G-protein-coupled CXC-motif receptor 4 (CXCR4). Stromal-derived factor-1 alpha (SDF-1α) is a well characterized, chemoattractant, regulating axon guidance and path finding preceding neuronal cell migration and guiding both neuronal and endothelial homing during organogenesis^39^,^40^,^41^,^42^. The alignment of our bioinformatics findings with extensive published data, led to the choice of the SDF-1α-CXCR4 ligand-receptor pair as candidates to validate our IPA predictions using molecular biologic and *in vitro* migration analysis techniques.

To validate the predicted effect of SDF-1α on PPC and RPC CXCR4 induced migration, we first used molecular techniques to define the presence and localization of the receptor. Next we used high-throughput Boyden chambers to establish optimal ligand concentrations for cell movement. Next, an engineered bridged μ-lane microfluidic device was utilized to generate mathematically defined SDF-1α gradients over long distances, allowing for time-lapse imaging of cell motility, polarization and directionality. Low concentration, stabile gradients guide cell migration during disease and development^43^,^44^ and biomimetic gradients can be well modeled using microfluidic technology^45^,^46^,^47^. Given the established spatiotemporal localization of SDF-1α and its receptor within the retinal microenvironment^42^,^48^,^49^, we utilized the bridged μ-lane to analyze PPC and RPC responses to SDF-1α with μ-mole gradient changes over distances comparable to the retinal microenvironment.

In summary, we present a comprehensive paradigm for defining ligand and cognate receptor interactions guiding migratory dynamics within a transplantation site. This approach begins with bioinformatics analysis of whole genome expression data from both the damaged retinal microenvironment and transplantable PPC populations. Data emerging from the bioinformatics stage, are validated using molecular and bioengineering approaches to provide a window into the complex signaling guiding PPC and RPC migration following subretinal transplantation for photoreceptor replacement. This approach is applicable to a range of retinal disease models to provide understanding of transplanted cell and host microenvironment communication.

## Results

### Migratory signaling pathways of transplanted PPCs in light-damaged adult retina

To identify migratory signaling networks activated during subretinal transplantation in light-damaged retina, ligands in the NSR and RPE were correlated with cell-surface receptors on rods and cones. Using IPA (Ingenuity^®^ Systems) bioinformatics platform, expression profiles were analyzed for the rod PPCs of Rho-EGFP mouse (P4, GEO Accession GSE29318)^50^, the cone PPCs of Bac‐Crx‐EGFP mouse (E17.5, GEO Accession GSE25607)^51^, and light-damaged mouse neurosensory retina and retinal pigment epithelia (10-week old, GEO Accession GSM928109)^35^. Ratios of mean signal intensity values, obtained from NSR, RPE and FACS-sorted PPC genome array results were uploaded to IPA software and converted to fold change values. After normalization, these values were used to generate statistical predictions of signaling governing cell migration.

Migratory signaling was modeled between NSR/RPE ligands and cognate PPC receptors with both significant expression levels and significant correlation to migratory ligand-receptor pairs in the IPA database. Next, a filter was applied requiring the involvement of each ligand and receptor in selected pairs to be involved in at least eight (8) out of ten (10) canonical, well established and characterized, cell movement subcategories listed in IPAs database (Supplemental Table 1a–d). Cell movement subcategories were ranked by p-value and predicted the effect of each genes expression state on activation or inhibition of each cell movement subcategory. This analysis yielded the following numbers of significantly predicted migratory ligand-receptor pairs: NSR/Rod PPC = 23, RPE/Rod PPC = 17, RPE/Cone PPC = 3, and NSR/Cone PPC = 3 (Table 1a–d). In Table 1, expression levels for each ligand and receptor are listed. Canonical activation or inhibition of migration subcategories for each ligand and receptor is based on interaction regardless of expression state. Predicted activation or inhibition correlates the expression levels of NSR/RPE ligands and PPC receptors in each pair to expression levels in IPAs database. The finding of inconsistent indicates a difference in expression levels between our dataset and expression levels for matched ligand-receptor pairs in IPAs curated database. Migratory ligand-receptor pairs with lower than 80% activation of cell movement subcategories are presented in Supplemental Table 2a–d.

In the NSR/Rod PPC dataset (Table 1a), canonical interactions lead to activation when fibronectin (FN1) binds to alpha-integrin (ITGAV) and RAC1. These signaling pathways are known to facilitate migration of human corneal epithelial cells^52^ and hepatocellular carcinoma cells^53^. However, the predicted interaction, based on down-regulated expression level of the receptors, compared to up-regulated fibronectin, is inconsistent with data in the IPA database. We observed that the down-regulated expression states of several migratory receptors on PPCs led to predicted interactions as inhibitory or inconsistent. The general down-regulated levels of PPC migratory receptors may contribute to limited migration following subretinal transplantation^19^,^31^,^54^,^55^.

The predicted effect of down-regulated PPC migratory receptor expression levels can be seen with α-integrin (ITGAV) and both inflammatory secreted phosphoprotein (SPP1) matrix metalloproteinase (MMP2), NOTCH1, and glial-derived neurotrophic factor (GDNF). Canonically, the GDNF ligand serves as a chemoattractant to GFRA1-expressing mouse GABAergic cortical cells^56^, rat glioma cells^57^,^58^ and mouse corneal epithelial cells^59^, and also serves neurotrophic functions in normal and diseased mammalian retina^27^,^60^,^61^. Additionally, upon ligand binding, GFRA1 requires secondary Ca^2+^-dependent activation of tyrosine kinase RET for downstream signaling^62^,^63^, demonstrating the significance of expression across signaling networks. Interestingly, CCL5, the T-cell specific RANTES protein, canonical and predicted interactions lead to activation of its cognate CCR3 receptor. Through literature review, we realized that although CCL5 is a known leukocyte [Table t2]chemoattractant and tumor cell motility factor^64^,^65^,^66^ its specific motility functions in neuronal cell types have rarely been defined.

Similarly, the RPE/Cone PPC dataset (Table 1d) predicts that VEGFA-KDR and MIF-CXCR4 ligand-receptor interactions canonically activate PPC migration, yet based on down-regulated expression levels inhibition is predicted. In the mammalian retina, VEGFA binds kinase insert domain receptor (KDR) expressed on retinal microvascular endothelial cells facilitating migration during hypoxia-induced neovascularization^67^. Given the down-regulated state of both the ligand-receptor pair, we may conclude with a high degree of certainty that only if VEGFA and KDR are up-regulated will PPC migration be facilitated. With an activating canonical interaction, it is probable that with up-regulation, macrophage migration inhibitory factor (MIF) and the C-X-C motif receptor 4 (CXCR4) will also lead to receptor activation influencing migration.

**Figure 1:**
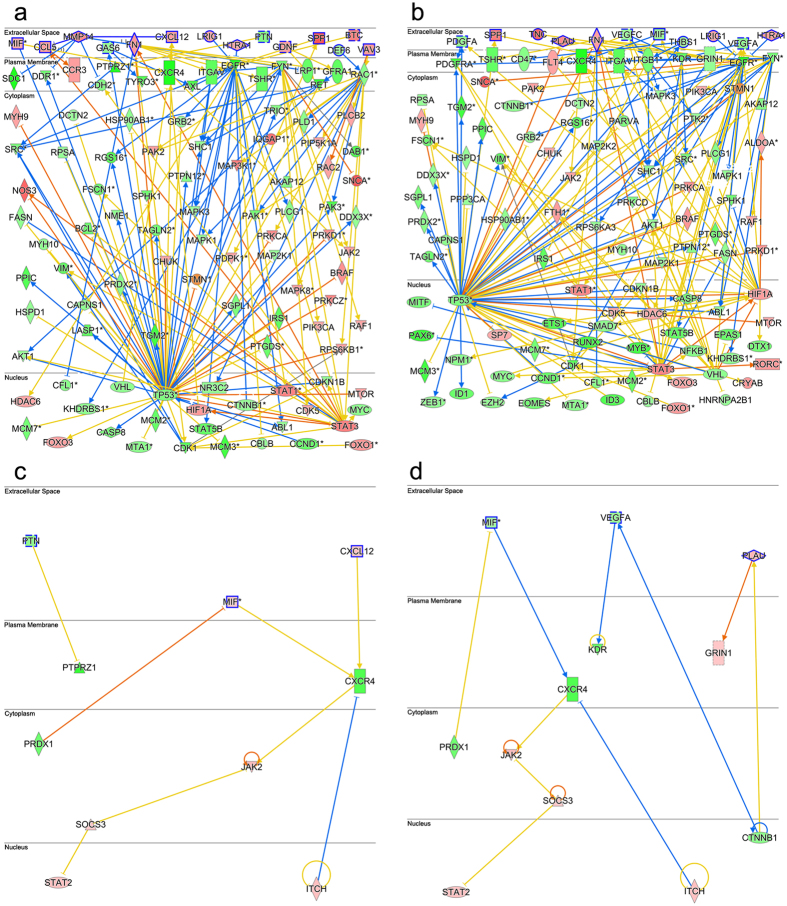
Ligand-receptor activated downstream signaling networks guiding PPC migration. Downstream network diagrams generated by pairing ligands from light damaged NSR and RPE to PPC cell surface receptors listed in Table 1. (**a**) custom network pathway for NSR/rod PPC, (**b**) RPE/rod PPC, (**c**) NSR/cone PPC and (**d**) RPE/cone PPC. Signaling molecules are compartmentalized into extracellular (ligands), plasma membrane (receptors), cytoplasmic and nuclear. Each lines connecting edge (arrow or bar) describes native interactions and line colors represent predictions, activation (orange line), inhibition (blue line) based on interacting gene expression states, up-regulated (red) or down-regulated (green). An inconsistent finding (yellow line) indicates a difference in expression level of the downstream molecule between our dataset and the IPA database relative to the expression level of the upstream gene. Canonical migratory network hubs in rod PPCs include STAT3 and TP53, comparable hubs in cone PPCs are STAT2 and ITCH.

To reveal downstream signaling of ligand-receptor interactions from Table 1, Fig. 1a,b represent custom network diagrams for the NSR/Rod PPC and RPE/Rod PPC datasets respectively. NSR/Cone PPC and RPE/Cone PPC datasets are displayed in Fig. 1c,d, respectively. Downstream networks were generated using the IPA molecular activity prediction (MAP) algorithm, which connected significantly expressed nuclear and cytoplasmic genes to migratory PPC receptor expression levels. PPC nuclear and cytoplasmic genes were required to be involved in 2 out of 4, p-value ranked, cell movement subcategories and their activation or inhibition states were assigned based on expression levels.

Ligand-receptor interaction networks in Fig. 1a–d are organized into extracellular matrix, plasma membrane, cytoplasmic and nuclear compartments. NSR-Rod PPC signaling in Fig. 1a reveals the active molecular hub, LDL receptor-related protein (LRP-1-dependent) and its endocytotic regulation of extracellular concentrations of matrix metalloproteinases (MMPs)^68^, leading to regulation of CXCL12 levels^69^. Also, signaling via the transcriptional regulator Notch 1 is active, a known regulator of cell fate and motility. Both LRP-1 and Notch 1 exemplify biochemical interactions predicted to be active in plasma membrane remodeling. Also, activation of Leukemia inhibitory factor receptor (LIFR) by its highly up-regulated ligand may lead to migration and neuroprotection as described previously in injured retina^70^. LIFR regulatory activity has also been reported to effect transcriptional and protein modification processes during retinogenesis^71^.

Network hubs present in both NSR and RPE Rod PPC downstream pathways (Fig. 1a,b) include the established regulators of migration, signal transducer and activator of transcription 3 (STAT3) and tumor protein P53 (TP53)^72^,^73^. Common nuclear elements between NSR and RPE cone PPC signaling pathways (Fig. 1c,d) include signal transducer and activator of transcription 2 (STAT2) and E3 ubiquitin-protein ligase Itchy homolog (ITCH), both associated migratory signaling^74^,^75^. This data demonstrates that downstream signaling pathways active in PPC migration are conserved across different tissue types and biologic processes. Ligand-receptor pairs, network pathways and hubs may be explored as molecular targets to activate or inhibit PPC migration within the retinal microenvironment.

### SDF-1α-CXCR4 interaction is predicted to guide migration of transplantable PPCs in light-damaged retinal tissue

Of the selected ligand-receptor pairs in Table 1 and Fig. 1, the interaction effects of stromal-derived factor-1 alpha SDF-1α binding to G-protein-coupled CXC-motif receptor 4 (CXCR4) interaction is of particular interest to our study. Both molecules are predicted to be significantly involved in the top ten cell movement subcategories identified using IPA. Also, being a canonical chemoattractant, SDF-1α has been shown to modulate axon guidance and migration across cell types^39^,^40^,^41^,^42^. While CXCR4 is down-regulated in the NSR/rod PPC and NSR/cone PPC datasets (Table 1a,d), SDF-1α exposure has been shown to increase receptor expression and activation. Several studies have reported SDF-1α induced CXCR4 expression in a range of cell types^76^,^77^,^78^,^79^. The effects of SDF-1α on CXCR4 dynamics involve phosphorylation, internalization and rapid desensitization^79^,^80^,^81^. Interestingly, neonatal CXCR4^+^ RPCs migrate towards tissues exhibiting higher SDF-1α concentrations during human retinogenesis and neovascularization^42^, suggesting a gradient-induced cellular response to the ligand. The SDF-1α ligand is released as an inflammatory cytokine during injury and increased expression levels have been reported following RPE NaIO_3_ chemical damage^82^ and irradiation^78^. Similarly, light-damage in our NSR and RPE sample resulted in up-regulated SDF-1α in the NSR/rod PPC (Table 1a) and NSR/cone PPC (Table 1c) datasets. IPA assigns statistical significance to the correlation of the SDF-1α-CXCR4 interaction and chemotactic migration of neuronal precursors^83^,^84^,^85^,^86^.

### NSR SDF-1α induced up-regulation of PPC-CXCR4 activates downstream migratory signaling pathways

**Figure 2:**
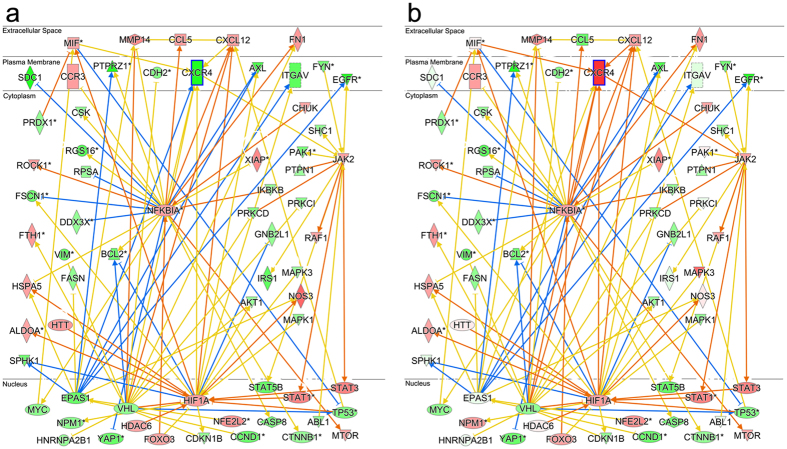
NSR SDF-1α - PPC CXCR4 downstream signaling pathway with receptor activation. (**a**) Using the NSR/rod PPC network pathway to highlight target interactions, downstream signaling cascades initiated via binding of candidate chemotactic factor SDF-1α to its CXCR4 receptor is shown to involve the canonical JAK-STAT pathway, which increases rates of migratory signaling molecule transcription^87^. Freshly isolated P4 rod PPC CXCR4 expression is down-regulated (green with bold outline) relative to other cells in the light-damaged retina and migration activation is supported as a canonical function. (**b**) With downstream signaling following CXCR4 up-regulation (red) via ligand exposure, migratory signaling activation emerges with expression levels comparable to other migratory cell types in IPAs database. Enhanced activation is observed between CXCR4 and JAK-STAT (orange arrow) as well as with MIF activation of CXCR4. Further downstream signaling events reveal the involvement of hypoxia-inducible factor-1 alpha (HIF-1α) and nuclear factor-kb (NFKB1A) in regulating transcription of the CXCL12-CXCR4 pair which increase chemotaxis in damaged neural tissue^89^.

To illustrate downstream signaling before and after up-regulation of PPC-CXCR4 by SDF-1α, released from the NSR, network diagrams were generated selecting for nuclear and cytoplasmic components specifically involved in half of IPAs top four cell movement subcategories. The cell movement categories include cell movement, migration of cells, cell movement of tumor cell lines, and migration of tumor cell lines. These categories were ranked by a p-value, correlating the effect of our nuclear and cytoplasmic gene set on the activity of each cell movement subcategory. Figures 2a,b show network pathways designed from datasets of NSR/rod PPCs with genes categorized by their subcellular localization.

In Fig. 2a, downstream signaling for the interaction of NSR released SDF-1α and PPC CXCR4 is highlighted and predicted to involve phosphorylation by Janus Kinase (JAK) providing docking sites for signal transducer and activator of transcription (STAT), which is eventually recruited to the nucleus to bind specific DNA promoters and increase transcription rates affecting a number of cellular processes, including cell migration^87^. However, this downstream JAK-STAT signaling is not predicted to occur in the network pathway given the down-regulated state of the CXCR4 gene in isolated P4 PPCs. The hypoxic conditioning of these freshly isolated cells may account for activated hypoxia-inducible factor (HIF1α) known to up-regulate CXCR4 mRNA in mesenchymal stem cells^88^. To demonstrate downstream signaling when the CXCR4 receptor is up-regulated, following SDF-1α exposure, we used a gene specification tool in IPA to exclusively up-regulate CXCR4 expression in this custom network pathway (Fig. 2b) and resolved signaling interactions 2-nodal steps away from the CXCR4 receptor (blue outline). Results highlight JAK-STAT as the major motility-deterministic signaling pathway for the CXCL12-CXCR4 ligand-receptor pair. Further downstream signaling events also reveal the cooperation of hypoxia-inducible factor-1 alpha (HIF-1α) and nuclear factor-kb (NFKB1A) in regulating transcription of the CXCL12-CXCR4 pair leading to PPC migration as predicted in our bioinformatics analysis and as reported elsewhere^89^.

### Boyden and microfluidic chemotactic screens confirm SDF-1α as a chemoattractant for PPCs and RPCs

**Figure 3:**
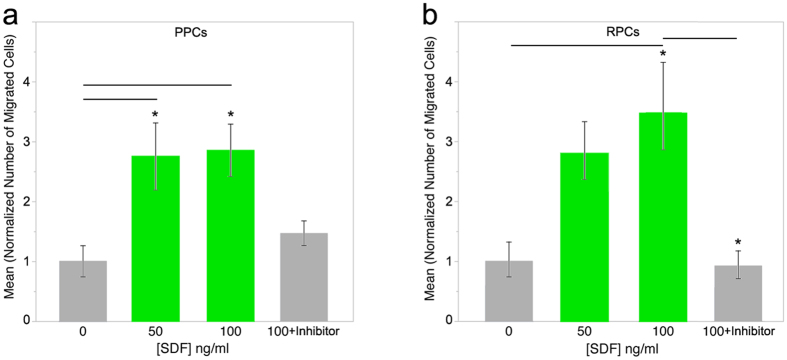
Boyden chamber analysis of PPC and RPC migration in uniform gradients of SDF-1α. Optimal SDF-1α concentrations were studied for induction of migration of isolated P4 PPCs and RPCs using the Boyden chamber. (**a**) PPCs displayed robust migration to SDF-1α at both 50 (*p = 0.0294) and 100 ng/ml (*P = 0.0193). 1 unit of normalization represents 87.3 cells. Following incubation in the CXCR4 inhibitor, AMD300, PPCs in 100 ng/ml SDF-1α gradients exhibited an observable decrease in rates of migration compared to 100 ng/ml SDF-1α alone. (**b**) SDF-1α chemokine induction of RPC migration appeared concentration-dependent and optimal at 100 ng/ml (*p = 0.0066). 1 unit of normalization represents 65.5 cells. AMD3100 inhibition of the CXCR4 receptor significantly reduced the number of RPCs migrating in 100 ng/ml SDF-1α gradients compared to SDF-1α alone (*p = 0.0238). Error bars depict mean ± SEM for three independent experiments. Statistical analysis was performed using ANOVA and post-hoc Tukey HSD.

To validate our bioinformatics modeling, we assessed the potential of SDF-1α to induce migration of transplantable PPCs and RPCs. To study optimal ligand concentrations, numbers of migrating cells and migratory dynamics in defined gradients quantitatively, two *in vitro* migration techniques were applied. Initial migratory analysis was performed using a modified Boyden chamber assay, which quantified transmembrane migration of PPC and RPC cell populations to SDF-1α gradients along a single axis, allowing for efficient characterization of optimal ligand concentrations. Next, mathematically defined, steady state micromole gradients of the ligand were generated using an engineered bridged μ-Lane microfluidic system, where single PPC and RPC responses could be correlated to specific gradient characteristics in a two-dimensional laminar matrix. Boyden chamber analysis showed that PPCs exhibited a robust migratory response to each SDF-1α concentration tested 50 ng/ml (*p = 0.0294) and 100 ng/ml (*P = 0.0193) (Fig. 3a). Boyden analysis of RPC migration comparing mean number of migrated cells in 50 ng/ml and 100 ng/ml SDF-1α revealed optimal concentration-dependent migration towards 100 ng/ml SDF-1α compared to control (p = 0.0083*) (Fig. 3b). The ligand concentration ranges used in these motility assays were also shown in previous studies to stimulate chemotaxis of retinal endothelial and cerebellar granular cells^48^,^90^,^91^.

### SDF-1α induces PPC and RPC chemotaxis via binding to cognate CXCR4 receptor

Next CXCR4 was inhibited to determine the degree to which interaction of the SDF-1α ligand with its receptor regulated the observed increase in ligand-induced PPC and RPC migration. CXCR4 expression has been shown to be necessary for the intracellular transduction of SDF-1α signaling during organogenesis, regeneration and migration of stem cells^89^,^92^. By analyzing inhibition of ligand-receptor binding in PPCs and RPCs, using the CXCR4 antagonist AMD3100, we assessed receptor-mediated SDF-1α chemotactic signaling specific to these transplantable cell types. Figure 3a,b show results of the AMD3100 inhibition of CXCR4 on PPCs and RPCs in the presence of 100 ng/ SDF-1α, respectively. Following pre-incubation with the receptor antagonist, PPCs showed a robust decrease in the mean number of migrated cells (F_(2,8)_ = 2.3; p = 0.1818) after AMD3100 treatment compared to 100 ng/ml SDF-1α control (Fig. 3a). Post-hoc Tukey HSD verified that significantly reduced numbers of RPCs migrated in the presence of 100 ng/ml SDF-1α +AMD3100 compared to SDF-1α control (p = 0.0364*) (Fig. 3b).

### Freshly isolated PPCs express CXCR4 while cultured RPCs require further induction by the SDF-1α ligand

**Figure 4:**
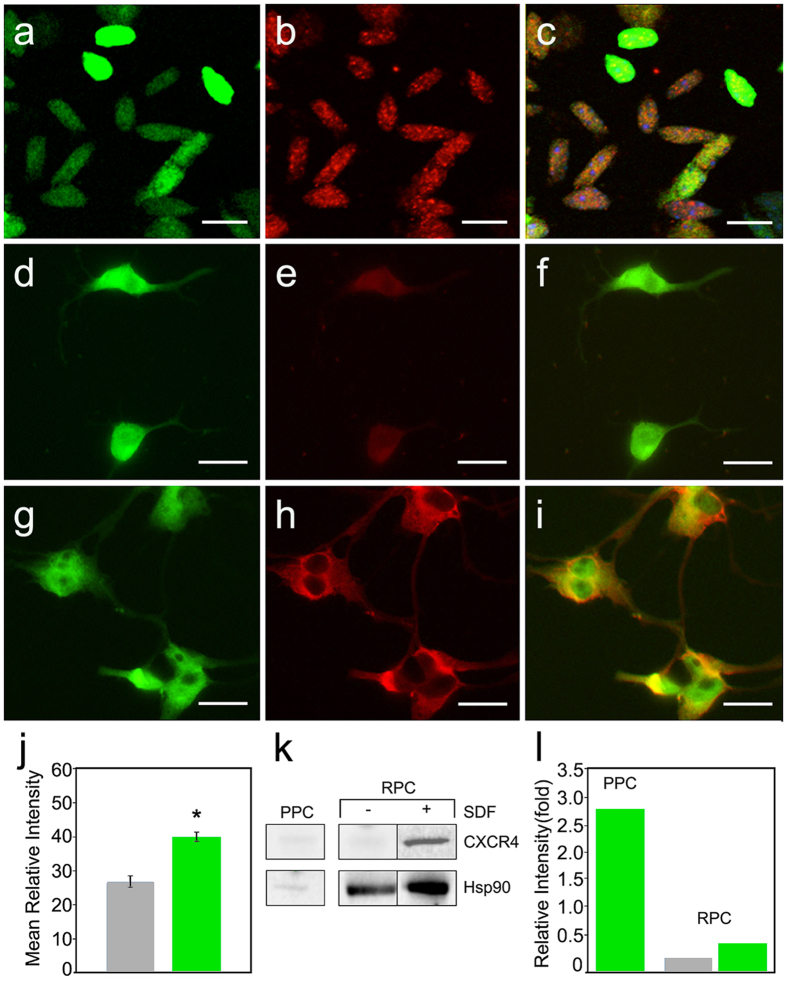
Heterogeneity in CXCR4 expression between transplantable PPCs and RPCs. (**a**) Wide-field fluorescence images of cytoplasmic GFP and (**b**) rhodamine bound antibody labeling of punctate SDF-1α receptors (CXCR4) constitutively expressed on freshly isolated P4 PPCs. (**c**) An overlay image of PPC GFP and rhodamine images, scale: 10 μm. In comparison, (**d**) cultured EGFP RPCs, (**e**) reveal minimal CXCR4-antibody bound TRITC fluorescence, (**f**) EGFP-TRITC overlay, scale: 20 μm. However, RPCs pre-incubated in 100 ng/ml SDF-1α exhibit robust CXCR4 localization on soma and along axonal processes visualized by TRITC conjugation to anti-CXCR4 (**g**–**i**) Scale: 20 μm. (**j**) Normalized pixel intensity values obtained for RPC confocal images of SDF-1α treatment, were significantly different (student’s T, p < 0.0001*) from control images (Error bars: ±SEM). (**k**) Western blot detection of CXCR4 protein in freshly isolated PPCs, non-treated and SDF-1α-treated RPCs. Light CXCR4 bands were detected in freshly isolated PPC lysates. Minimal CXCR4 expression appeared in control RPCs with significant increase in receptor presence following SDF-1α exposure. Heat-shock-protein 90 (HSP90) was used as loading control. (**l**) Relative intensity analysis of western blot bands normalized to control (HSP90) for PPCs and RPCs separately. Primary isolated PPCs exhibit CXCR4 levels above control following background subtraction. Cultured RPC CXCR4 levels were slightly detectable initially and exhibited a moderate increase following exposure to SDF-1α.

To verify the presence and localization of the receptor, immunofluorescence detection of CXCR4 was performed on PPCs and RPCs. Anti-CXCR4 antibody detected innate constitutive expression of the receptor on freshly isolated PPCs, which appeared localized across the surface of the oblong-shaped cells (Fig. 4a–c) consistent with immature rod morphology^17^,^30^. RPCs showed minimal CXCR4 staining in the absence of SDF-1α exposure (Fig. 4d–f) and following incubation overnight with 100 ng/ml SDF-1α (Fig. 4d–f) exhibited visible increases in receptor expression on soma and along branching processes (Fig. 4g–i). RPC anti-CXCR4 conjugated TRITC images taken from control and SDF-1α -treatment were analyzed for mean intensity values as a measure of cell surface receptor density and RPCs incubated in 100 ng/ml exhibited significantly higher receptor densities compared to control (Fig. 4j, p < 0.0001). The heterogeneity in receptor expression between PPCs and RPCs is likely the result of the ontogenetic state of the cells and the freshly isolated conditions of PPCs compared to the cultured conditions of RPCs. Following isolation of PPCs, western blot analysis of cell lysate revealed the presence of the CXCR4 protein (Fig. 4k). Upon stimulation with the ligand, western blot analysis of RPC lysates reveals a distinct band of CXCR4 not detectable in control (Fig. 4k). It is plausible that the process of PPC isolation leads to up-regulation of the CXCR4 receptor as an inflammatory response. In addition, physiologic SDF-1α levels are present in the mouse retina^93^ and comparable levels have been shown to induce cell surface receptor expression on a number of cell types through phosphorylation and priming mechanisms^79^,^81^. In contrast, cultured RPCs have not been exposed to the SDF-1α ligand over successive proliferative cycles.

### Nanamole SDF-1α gradients stimulate migration of PPCs and RPCS in an engineered microfluidic system

**Figure 5:**
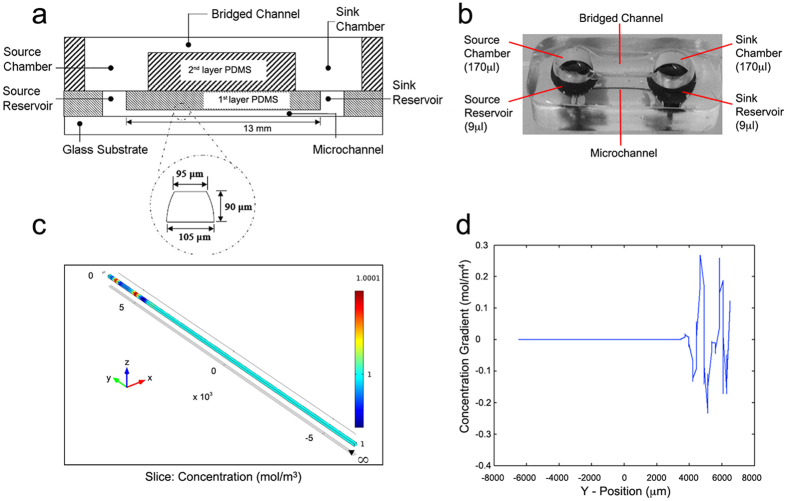
The Bridged μ-Lane microfluidic system generates steady state chemical gradients of chemotactic factors. (**a**) Schematics of the bridged u-lane shows the first layer PDMS with source and sink reservoirs linked through a microchannel measuring 13 mm (length), 90 μm (depth), 100 μm (width), and 95 μm (hydraulic diameter). The second layer PDMS, the user interface layer, is a semi-circular open bridged channel that measures 9 mm (length) and 2 mm (depth) and fluidically connects to the first layer via source and sink chambers. (**b**) Functional bridged u-lane microchannels are fabricated by soft lithography of PDMS on dimensioned aluminum casts followed by polymer-polymer bonding of first and second PDMS layers and then bonding of PDMS to glass. Mass differences between drop-wise loaded motogens in source chambers and media control in sink chambers induces diffusive chemical flow through the microfluidic system with minimal hydrostatic pressures because the bridged channel maintains fluid volume equilibration. (**c**) 3-D COMSOL model of SDF-1α (mol.wt. ~7.9 kDa) diffusivity in water along the Y-axis length of the microchannel from infinite supply of 1M SDF-1α in SRR (x,6500 μm,y) to 0M SDF-1α in SKR (x,−6500 μm,y). (**d**) 1-D plot of changing SDF-1α concentration with increasing distance from SRR to SKR loci (8000 ≫ −8000 μm). SDF-1α concentration profile after the first few thousand microns (~2700 um) is unchanging and two-dimensional transport models of epidermal growth factor (EGF, mol.wt. ~6.0 kDa) and 10 kDa Dextran concentration previously assessed in the bridged μ-Lane^95^,^96^ established sustained SDF-1α gradients between 18–40 hrs after introduction of the ligand into the microfluidic system.

**Figure 6:**
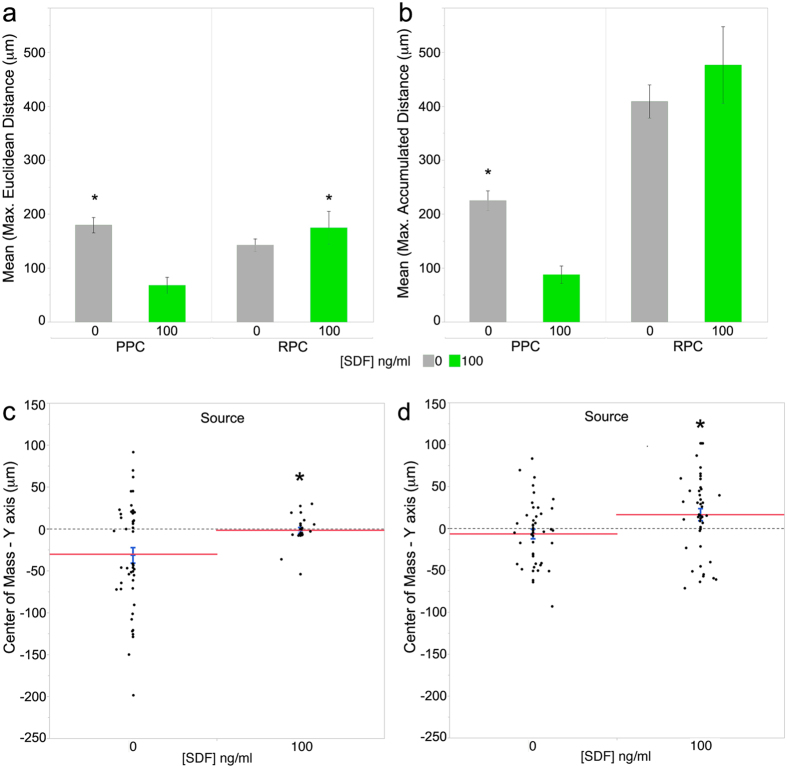
PPC and RPC migratory dynamics in an engineered microfluidic SDF-1α gradient. (**a**,**b**) PPCs in control microchannels traveled longer accumulated and Euclidean distances, respectively, compared to cells in SDF-1α gradients. (**a**) RPC accumulated distances were significantly longer in the presence of the ligand and (**b**) Euclidean distances were comparable to controls. The center of mass (COM), spatial averaged endpoints, for (**c**) PPCs and (**d**) RPCs after 24 hrs in SDF-1α gradients showed significant migration towards the source ligand reservoir compared to both cell types in control (PPCs P = 0.0033, RPCs P = 0.0135). These results indicate that PPC and RPCs are exhibiting ligand-directed chemotactic migration. All assays were carried out in triplicates. Each blue/black error bar is constructed using 1 standard error from the mean. Asterisks denote significant mean difference in pairwise comparison between experimental control conditions. Table 2 displays descriptive statistics for the microfluidic assay including the number of cells tracked and values of motility parameters measured.

The bridged μ-Lane microfluidic system, a type of flow-resistive gradient generator^94^, maintains distinct regions of constant ligand concentrations that form time-invariant gradients along the microchannel. Ligands travel down the channel by diffusive transport caused by mass differences between ligand source and sink reservoirs, while minimizing fluid convection^95^. Using mathematical models of similarly weighted epidermal growth factor (EGF, 6.8 kDa) and 10 kDa dextran transport within the bridged μ-Lane^95^,^96^ we verified that the duration of SDF-1α steady state gradients would range from 18 to 40 hrs after ligand addition into the source reservoir (methods, Fig. 5a–d) . Fig. 6a,b show that while PPCs exposed to steady state SDF-1α gradients traveled significantly shorter distances compared to control conditions (Max. Euclidean: t_(60.26)_ = −5.37, p < 0.0001*; Max. Accumulated: t(_66.92_) = −5.63, p < 0.0001*), their spatial averaged end-points (center of mass (COM)) was significantly displaced towards the positive Y-axis, containing higher ligand concentration gradients (Fig. 6c, COM-Y-axis: t_(57.91)_ = 3.06, p = 0.0033*). Similarly, RPCs migrated towards increasing concentrations of SDF-1α, with significantly higher maximal Euclidean distance (Fig. 6a, t_(83.1)_ = 2.29; p = 0.0247*), and mean COM Y-axis (Fig. 6d, t_(82.25)_ = 2.52; p = 0.0138*) compared to cells in control microchannels. The COM is a major index for evaluating directed chemotaxis with either positive or negative coordinates indicating the direction in which the population of cells have migrated, and a magnitude that measures the difference between the cell population COM at the beginning and at the end of the experiment^97^.

**Figure 7:**
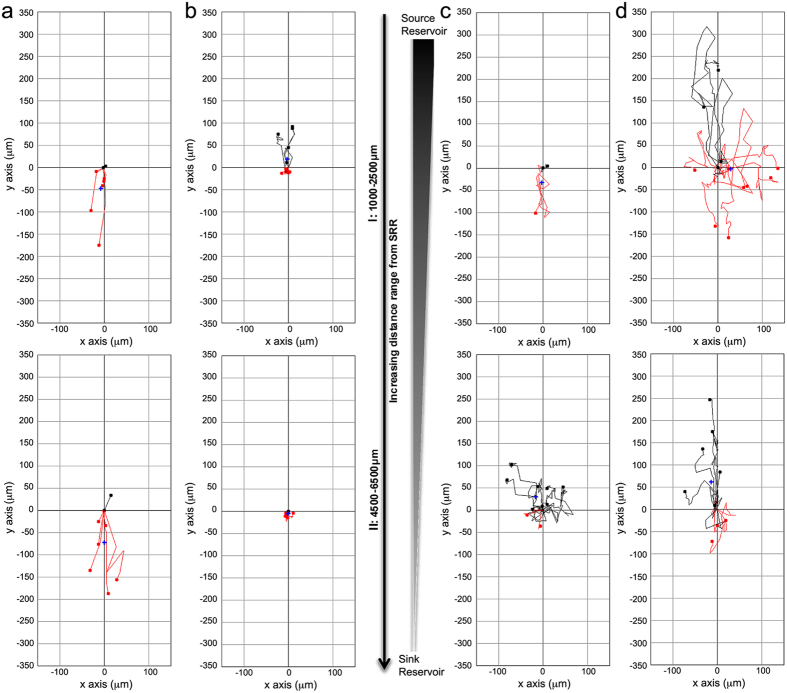
Wind-Rose plots of PPC and RPC migratory dynamics in microfluidic gradients of SDF-1α. PPC and RPC **SDF-1α** gradient induced migratory dynamics were imaged over a 24 hr period, in13 mm microfluidic μ-lane devices. PPC and RPC movements were tracked and are depicted in wind-rose plots at increasing distances from the source reservoir (SRR): 1000–2500 μm (I), 4500–6500 μm (II). (**a**) PPC migration in control conditions shows random and increased non-directed migration. (**b**) In 100 ng/ml SDF-1α steady-state gradients PPC exhibit significant directed migration toward the SDF-1α source. (**c**) RPC dynamics in the absence of the SDF gradient (control) resulted in increased random Euclidean movement away from the ligand source. (**d**) In the presence of the100 ng/ml SDF-1α gradient, both RPCs and PPCs show significant difference in their center of mass (COM) toward the Y-axis source of SDF-1α (Fig. 6: PPCs p = 0.0033, RPCs p = 0.0135). Red and black traces indicate cells with negative (away) and positive (toward) COM, relative to the ligand source. The X- and Y-axis denote cell displacement in horizontal and vertical directions, respectively. All measurements of migration dynamics were performed between 18–42 hrs, in the presence of steady state nanomole SDF-1α gradients. Complete descriptive statistics of the trajectory plots depicted here are reported in Table 2.

To visualize patterns of cell movements detected using live-imaging in microfluidic systems, representative trajectory plots of chemotactic migratory dynamics at increasing distances (1–6.5 mm) from the SDF-1α source reservoir are presented for PPCs Fig. 7a,b and RPCs Fig. 7c,d. The top plots in Fig. 7a–d represent cells tracked within 1000–1250 um from ligand (6b,d) or control source (6a,c) wells, while bottom graphs represent tracking between 4500–6500 um from source wells. At 1000–1250 um from ligand source both PPCs and RPCs (6b,d, respectively) exhibit greater overall migratory activity compared to controls. It can also be observed that there are both random and directed migratory dynamics across cell trajectories in different conditions.

## Discussion

This work demonstrates that bioinformatics modeling and microfluidic testing can begin to explain the complex molecular signaling influencing PPC and RPC migratory responses within the damaged retinal microenvironment. The uniqueness of this approach lies in the fact that the gene expression states of selected ligand-receptor molecules were used to characterize signaling to make determinations about downstream effects on transplantable PPC and RPC migration. In addition, using microfluidic techniques, the influence of an individual ligand can be described in mathematically defined gradients to characterize specific cell migration properties.

This study focuses on identifying molecular signaling guiding migration of transplantable PPCs in a light damaged retina model. A similar bioinformatics paradigm could provide data describing signaling guiding human iPSC derived PPC migration in pathogenic retinal environments, such as age-related macular degeneration and retinitis pigmentosa. Additionally, bioinformatics could help define down-stream networks involved in vector-mediated gene therapy^27^,^98^,^99^ and stimulation of endogenous progenitor cell dedifferentiation toward repopulation of photoreceptors^100^,^101^. A limiting factor with gene-therapies is the genetic heterogeneity characterizing many retinal diseases^99^ and the activation of endogenous progenitors remains limited due to restricted differentiation potential. A comprehensive bioinformatics approach may help characterize molecular targets to be modulated toward improved outcomes.

In this work the top migratory signaling pathways between NSR and RPE ligands and cognate PPC receptors are presented based on expression levels and correlation to published migration data in the IPA database. This analysis suggests that molecular mechanisms involved in PPC motility are conserved across a range of cell types. It was observed that a number of PPC migratory receptor genes are down-regulated compared to their up-regulated cognate ligands in damaged NSR and RPE. It is possible that during initial transplantation, down-regulated PPC migratory receptors may not detect ligands present in the retinal microenvironment leading to reduced migration rates. Based on data presented here, that SDF-1α exposure increases CXCR4 expression in RPCs, pre-incubation of transplantable cell populations with ligands known to be expressed in the retinal microenvironment may up-regulate receptor expression and facilitate migratory signal detection. Both PPC and RPC receptor-ligand signaling and downstream signaling pathways can be examined further to better understand and enhance activation of migratory processes during transplantation^102^,^103^.

To test the bioinformatics generated ligand-receptor data, the role of SDF-1α was verified as a migratory chemoattractant for both PPCs and RPCs via activation of its cognate CXCR4 receptor. Boyden and microfluidic analysis demonstrated that signaling downstream of CXCR4 receptor activation led to significant directed PPC and RPC migration toward the ligand source in uniform and steady state gradients. This represents an initial step in defining the influence of top predicted NSR and RPE ligands interacting with cognate PPC receptors to define migratory response dynamics. Additional ligand-receptor pairs resolved using bioinformatics in this study may serve as targets for future validation studies. Future studies may also evaluate three-dimensional matrix gradients and multiple interacting soluble ligands, to develop retinal biomimetic migration chambers, informed by retinal microenvironmental ligand data. Data provided here may enhance future photoreceptor replacement strategies by providing molecular targets to help modulate migration and optimize outcomes of PPC transplantations. This comprehensive approach to defining communication between a transplantation site and a transplantable cell population may also be applied to a range of CNS and other tissues under investigation for cell transplantation therapy.

## Materials and Methods

### Microarray based IPA Bioinformatics Analysis

Transcript arrays of rod (N = 38626) and cone (N = 20744) precursors annotated in the IPA knowledgebase were identified as mapped IDs, also termed network eligible (NE) molecules. NE molecules were similarly resolved from transcript IDs of neurosensory retina (NSR) and retinal pigment epithelium (RPE) of light damaged adult retina (N = 23176). Following the subcellular localization of NE molecules to their ECM, plasma membrane, cytoplasmic or nuclear loci, and a statistical cut-off for genes whose resolved mean intensity values are significantly greater than their negative FAC-sorted analogue by a t-test p-value of 0.05, NE extracellular matrix (ECM) molecules resolved from the NSR and RPE dataset were then matched with NE plasma membrane receptors from the rod and cone photoreceptor microarray dataset. Matched pairs included – 1) rod receptor genes matched with ECM genes from NSR (N = 2013), 2) rod receptor genes matched with ECM genes from RPE (N = 1945), 3) cone receptor genes matched with ECM genes from NSR (N = 735), and 4) cone receptor genes matched with ECM genes from RPE (N = 667). These matched pairings between molecule sets define biochemical interactions between receptors on transplantable photoreceptor precursor cells (PPCs) at their sub-retinal grafting site, and existent ECM ligands released into the retinal microenvironment upon light damage to the neurosensory (NSR) and retinal pigment epithelial regions.

**Figure 8:**
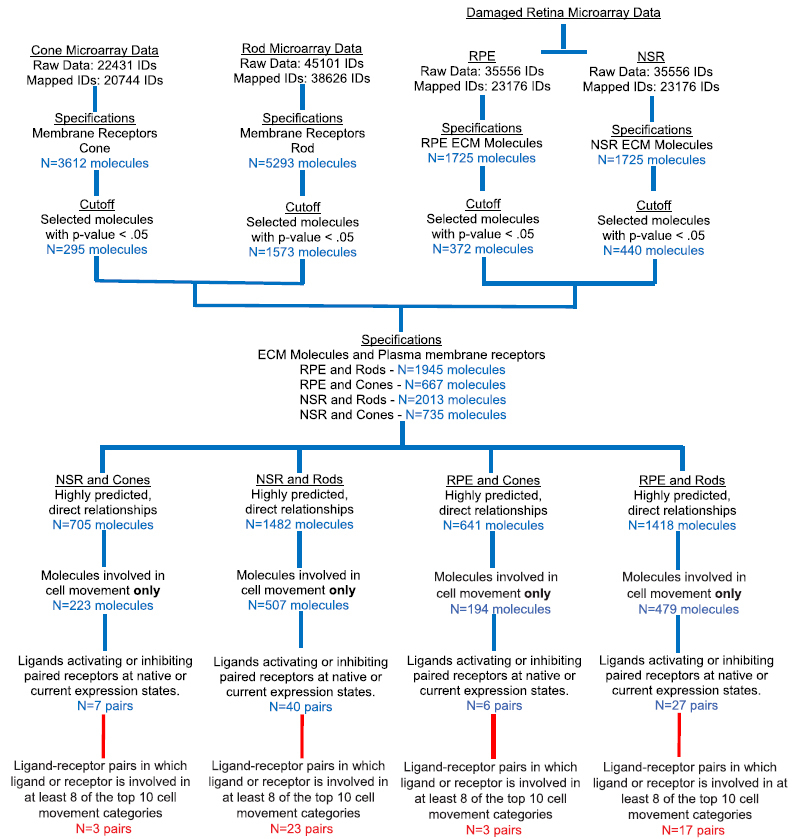
Bioinformatics analysis of damaged retina and PPC microarray datasets. Schematic of workflow for selecting genes significantly predicted to guide migration of transplantable PPCs in a light-damaged photoreceptor degeneration model. Whole genome NSR, RPE and PPC microarray gene intensity values were assessed for significant difference in their expression states (p <0.05), and ratios of positive/negative intensity values of selected genes were uploaded to IPA as fold change values. Selected genes were then paired to mimic typical sub-retinal transplantation paradigms: Cone and rod PPC receptors paired with extracellular ligands from light-damaged NSR and RPE. A core analysis of each pairing mapped constituent genes to their occurrence in IPA knowledgebase based on chance alone using a Fisher Exact test. Genes involved in cellular movement were selected and used to design network pathways that take into account for the expression level of each gene and their effects on cellular movement. Ligand-receptor pairs whose expression states predict an activating or inhibiting effect on cell movement, or whose effect on cell movement is inconsistent with literature in the IPA database are selected and their involvement in the top ten cellular movement subcategories as well as their individual pairwise effect in each relevant subcategory were used as criteria to determine candidate chemotactic ligand-receptor pairs for *in vitro* cell migration assays. Intracellular PPC downstream signalling pathways following candidate receptor activation were resolved to model statistically predicted plasma membrane, cytoplasmic and nuclear molecules involved in cell response properties and migratory dynamics.

After IPA core analysis of the four matched data sets, molecules in each dataset mapping to cellular movement functional clusters were used to design signaling networks of direct interactions between ligand and receptor pairs based on their gene activity states, predicting an activating or inhibitory relationship, or a relationship inconsistent with the expected gene interaction curated in the IPA knowledgebase based on the state of the downstream gene in a matching gene pair. In performing the core analysis function on our matched datasets, the IPA network algorithm uses gene identifiers in our data to build small networks based on their interconnectivity and to molecules they connect to in the IPA knowledgebase. A right-tailed Fisher exact test (p < 0.05) is then used to calculate the probabilistic fit between the networks and lists of biological functions and canonical signaling pathways curated in the IPA knowledgebase, assigning scores to networks based on the probability of associating network molecules to gene annotations in the IPA by random chance only [83]. A stepwise flowchart of bioinformatics selections criteria is provided here in Fig. 8.

Genes were resolved co-linearly with corresponding fold-change values specific for each cell type in each matched tissue dataset. In the subsequent matched dataset analysis, we used IPA to select for only PPC plasma membrane receptors and direct matching ligands from NSR and RPE that direct cell movement (Table 1). Given our objective of further refining motility-deterministic signaling between ECM ligands and target receptors on PPCs, pairs correlating to cell motility in each data set were selected for an IPA Downstream Effects analysis to statistically correlate gene expression states to cell motility, predicting an increase or decrease in function based on direction of fold change values of genes in our data sets (Fig. 2). Subsequently, custom pathways for selected genes in each matched dataset pair were used to generate a library of ECM molecule and PPC receptor interactions that govern cell motility in damaged adult mouse retina. We then used the IPA database to correlate canonical migratory signaling pathways to cell motility ligand-receptor networks derived from our datasets.

### Retinal Cell Dissociation and Culture

All animal procedures were performed in compliance with the Association for Research in Vision and Ophthalmology (ARVO) statement for the use of animals in ophthalmic and vision research, and were approved by the City University of New York, Lehman College Animal Care and Use Committee (IACUC). Mice cone-rod homeobox (Crx) promoter driven GFP (Crx/GFP) on a C57BL/6J background (Jackson Labs) were maintained at the Lehman College Animal Facility. Photoreceptor precursor cells (PPCs) were isolated as previously described^50^,^104^,^105^ from eyes of post-natal day 4 (P4) Crx/GFP^+/+^ pups. Enucleated eyes were immediately submersed in 70% ethanol and then suspended in cold Neurobasal medium (NB). Next, an incision was made at the ora serrata and the neural retina was separated from the sclera and lens using forceps. Detached retina were warmed at 37 °C for 8 min in pre-warmed (37 °C) trypsin (0.05%) (Sigma-Aldrich, St.Louis, MO) in Hanks Balanced Salt Solution (HBSS) (Life Technologies, Grand Island, New York) for 20 min. Trypsinization was inhibited using FBS (20%) and DNase I (0.2%) in HBSS. Cells were dissociated by trituration in 1% BSA, 10 mM HEPES and 2 mM EDTA HBSS solution and passed through a 40 μm pore diameter filter to remove clumps and artifacts. Cells were resuspended in Ca^2+^- and Mg^2+^-free HBSS and put on ice until use. For RPCs, dissociated retina from P4 mice with beta actin-promoter driven EGFP^+/+^ on a C57BL/6J background (Gift from Dr. Young, Harvard University), were expanded and culture-maintained at 37 °C/5% CO_2_ in mitotic NB complete medium containing 2% B-27, 1% L-glutamine, 1% Pen Strep, 1% N2 (50X), 2% Nystatin, 93% NB only (Invitrogen-Gibco, Rockville, MD), 20 ng/ml epidermal growth factor (Promega, Madison, Wisconsin) as previously described^106^.

### Immunocytochemistry

Freshly dissociated PPCs were allowed to adhere to gelatin-coated (0.5%, Sigma-Aldrich, St.Louis, MO) glass slides (n = 6), fixed with 1% PFA in PBS for 10mins and briefly passed over 35 to 45 °C heat. RPCs from culture were fixed for 10mins in 1% paraformaldehyde in PBS on coverslips (15 mm (19/32″), Thermoscientific, Rochester NY). Some RPCs were also pre-incubated in 100 ng/ml SDF-1α overnight at 37 °C/5% CO_2_ prior to fixation. Cell staining procedures were performed as previously described^107^,^108^. Briefly, cells were rinsed in wash buffer (0.1% bovine serum albumin (BSA) in PBS, non-specific staining was blocked and cells permeabilized with 0.3% Triton^®^ X-100 and 10% normal donkey/goat serum (Sigma-Aldrich, St. Louis, MO) for 45 minutes at room temperature. Rabbit polyclonal (1:500, Abcam Ab7199, Cambridge, MA), and rat monoclonal (1:50, R&D systems Mab21651, Minneapolis, MN) anti-CXCR4 primary antibodies were diluted in incubation buffer (1% BSA, 1% normal donkey/rabbit serum, 0.3% Triton X-100 and 0.01% sodium azide in 1X PBS) and added to PPCs and RPCs respectively for overnight incubation at 4 °C. Incubation buffer was added to cover slips as negative control. After 3–5 washes with wash buffer, coverslips with RPCs were then incubated with NL637 fluorochrome-conjugated Goat-Anti-Rat (1:200; R&D systems, Minneapolis, MN) secondary antibodies, while PPCs were incubated in Dylight 594 Goat-Anti-Rabbit (1:500; Jackson ImmunoResearch, Westgrove, PA) for 1 hr at room temperature. After rinsing once for 15 min and thrice for 5 min with wash buffer, cover slips were inverted onto glass slides coated with DAPI-containing anti-fade mounting medium (Life Technologies, Grand Island, NY). DAPI-stained nuclei, GFP^+^ cell body and TritC-conjugated CXCR4 expression were assessed on an inverted fluorescent microscope (Nikon Eclipse Ti, Melville, NY) using 40X and 100X objective lenses. Confocal images (Nikon C1 Digital Eclipse Modular Confocal Microscopy System) of stained SDF-1α-treated and non-treated PPCs and RPCs were also obtained in triplicates for use in calculating net integrated density values of selected cell image views with Trit-C filter on an image J platform after subtracting background mean gray values. In this procedure, pixel intensity and density over the selected image area served as a semi-quantitative assessment of cell surface CXCR4 expression due to SDF-1α effects on RPCs. A statistical comparison of mean integrated density values of the two treatment groups was then performed using a student’s T-test^109^.

### Western Blot Analysis

Cell lysates were prepared in ice cold lysis buffer (0.5ml per 5 × 10^6^ cells) containing: 150 mM NaCl, 1% Triton X-100, 50 mM Tris, pH 8.0, 1 mM phenylmethylsulfonyl fluoride (PMSF, Sigma-Aldrich, P7626), Halt Protease inhibitor Cocktail (Pierce IL, 78425), and phosphatase inhibitor (Sigma-Aldrich, P5726) with RPCs that had been incubated for 24 hrs with 0 ng/ml or 100 ng/ml SDF, and to freshly dissociated Crx-GFP PPCs sorted using a BD FACS Jazz. The GFP-positive cells were sorted at 4,000 cells per second in 1.5-drop pure mode and collected into 3ml tubes. RPC and sorted PPC mixtures were agitated for 30mins and centrifuged at 12,000rpm for 20mins at 4 °C. The resulting supernatant was used to determine protein concentration by the Bradford assay and subsequently, 25–40 μg of protein was separated on 8% SDS-PAGE (Clear Page Gel, CBS Scientific, San Diego, CA) and then transferred onto nitrocellulose membranes. The membranes were probed overnight at 4 °C with monoclonal rat anti-CXCR4 (1:500, R&D systems Mab21651, Minneapolis, MN) for PPCs and polyclonal rabbit-anti-CXCR4 (1:1000, SAB3500383 Sigma-Aldrich, St. Louis, MO) for RPCs. The membranes were probed with secondary antibodies conjugated to horseradish peroxidase (HRP) (RPCs - 1:1000, CST 7074S anti-rabbit IgG, Cell Signaling Technology, Danvers, MA; PPCs – 1:1000, Polyclonal goat anti-rat HAF005 R&D Systems) for 1 hr at room temperature and detection was carried out after applying enhanced chemiluminescence substrate mixture (ECL Plus; Pierce IL). Heat shock protein HSP90 (1:1000, #4874 Cell Signaling Technology) was used to correct for blotting efficiency and normalization. Standard molecular weight markers (Bio-Rad Laboratories Inc.) served to verify the molecular size of CXCR4 at 47 kDa and of HSP90 at 95 kDa^76^,^110^. Western blot bands were measured with ImageJ software and normalized to controls for PPCs and RPCs separately.

### Boyden Chamber Migration Assay and Motility-inhibition studies

RPC and PPC chemotaxis to a uniform gradient of stromal derived factor (SDF-1α) was assessed using a modified Boyden chamber assay as previously described^90^,^111^. Serum starved RPC cultures and fresh PPC isolates were pelleted and re-suspended in NB media supplemented with 10% fetal bovine serum (FBS), at a seeding density of 5 × 10^4^ cells/350μl volume, in the upper chamber of non-coated polyethylene terephthalate (PET) membrane filters (8μm pore size, BD Falcon, NJ). Filters were inserted into tissue culture wells (BD Falcon, NJ) containing 700 μl volumes of NB media containing 0, 50 or 100 ng/ml SDF. Triplicate wells per treatment were incubated for 24 hrs at 37 °C/5% CO_2_. For AMD3100 inhibition studies (Sigma-Aldrich, St. Louis, MO), both PPCs and RPCs were pre-incubated with 5 μg/ml of the antagonist for 30mins at 37 °C and 5% CO_2_ before loading in the upper chamber as previously described^76^,^112^,^113^. After 24 hrs, filters were fixed with 4% paraformaldehyde in PBS for 5 min and stained with diluted (1:1000) Hoechst 33342 nuclear stains for 10 min. Non-motile cells were scraped from the upper chamber, and the number of stained migrated cells at the bottom surface of the filter was resolved by counting 5–10 view-fields per filter for the triplicate filters in three independent experiments using an inverted fluorescent microscope. Control tissue culture wells contained NB media without any added chemotactic factor (migration study) or without the chemotactic factor and/or inhibitor (inhibition study). ANOVA (F) and pairwise Tukey HSD statistics assessed significant mean differences (p < 0.05) in the number of transmigrated cells normalized to chemoatxis in control filters.

### The Bridged μ-lane Microfluidic System

Our engineered microfluidic device, the bridged μ-lane was employed to generate mathematically defined chemical gradients for imaging of PPC and RPC behaviors. Two-dimensional ligand mass transport within the bridged μ-lane was computed using the constitutive relations of continuity, convective–diffusion, momentum equation and hydrostatics on a finite element methods platform (FEMLab Version 3.4, Comsol Inc., Burlington, MA), which generated models of gradient profiles^95^,^96^,^114^. This microfluidic device does not require perfusion, maintaining steady state gradients for up to 72 hrs for self-contained assessment of cellular motility.

### The Bridged μ-lane Microfluidic System Fabrication

The bridged μ-Lane device consists of two layers of silicon polymer poly-dimethylsiloxane (PDMS): a closed microchannel (0.1 μL volume, 95-μm-hydraulic diameter: 90 μm-depth, 100 μm-width; 1.3 cm-length), a source reservoir (SRR) and a sink reservoir (SKR) (9 μl volume each) on the bottom layer, and a source chamber (SRC) and sink chamber (SKC) (170 μl volume each) connected by an open, hemispherical bridge channel (2-mm-depth; 9-mm-length) on the top layer (Fig. 5a). The device is fabricated using elastomeric molding of PDMS and bonding of PDMS to PDMS and PDMS to glass. The SRC and SKC in the top layer are fluidically connected to the SRR and SKR in the bottom layer, and the bridge channel connects the SRC to the SKC in order to balance their solution volumes (Fig. 5b). The complete bridged μ-Lane system is thus composed of the upper user interface layer with an open bridge channel that connects the SRC and SKC chambers, as well as a bottom layer closed microchannel that connects the SRR and SKR reservoirs^95^. The double-layered PDMS is then bonded to chemically cleaned (Nanostrip, Freemont, CA) glass slides using ozone gas exposure. Cells are incubated along the microchannel and the cell culture media is manually loaded until it has filled the SRR, microchannel, SKR, SKC, and bridge channel. The engineering design of the bridged μ-lane uses the large chamber/reservoir, reservoir/microchannel and chamber/microchannel volume ratios to facilitate sample loading via conventional pipette to maintain constant reagent concentration and gradual fluidic transport into the microchannel over appropriate experimental timescales. The bridged channel eliminates hydrostatic pressure differences between the SRC and SKC so that only density differences between SRC and SKC reagent concentrations exist and drive convective minuscule bulk velocity flow through the microchannel in the bottom layer, while minimizing time required to attain a steady-state gradient of the ligand^95^,^96^. The test chemical solution is micropipette loaded into the SRC until the sample makes contact with the solution within the bridge channel to initiate system operation.

### Computational modeling of SDF gradient evolution

Experimental measurement of SDF-1α (mol.wt. ~7.9 kDa) transport within the bridged u-Lane in this study was computed using the multi-physics modeling software COMSOL (COMSOL Inc., Burlington, MA). In the analysis, we assume chemical species diffusion through water from an infinite 1M reservoir (SRR) to sink (SKR) at either side of the microchannel with the entire system governed by a standard transport of diluted species model within COMSOL. Figure 5c displays a 3D model of SDF diffusivity along the Y-axis length of the channel from SRR coordinates (x,6500 μm,y) to SKR coordinates (x,−6500 μm,y). In Fig. 5d we present a one-dimensional plot of changes in SDF-1α concentration gradient with increasing distance from SRR to SKR loci (8000 ≫ −8000 μm). Within the accuracy of the model, the SDF-1α concentration profile after the first few thousand microns (~2700 um) is unchanging^115^. Time-dependent attainment of SDF-1α steady gradients models two-dimensional transport of similarly weighted epidermal growth factor (EGF, mol.wt. ~6.0 kDa) and 10 kDa Dextran concentration previously assessed in the bridged μ-Lane^95^,^96^. In these investigations, steady state gradients of EGF and Dextran measured as a function of time and axial position, were established after 18 hrs and 40 hrs of microchannel incubation respectively, and given the diffusion coefficient of SDF-1α ~ 1.11–1.7 × 10^−6^ cm^2^/s^116^,^117^,^118^ within the range of EGF and dextran values (0.82–2 × 10^−6^ cm^2^/s), evaluation of cell response to sustained SDF-1α gradients was carried out between 18–40 hrs after introduction of the ligand into the microfluidic system.

### Microfluidic Assay and Microscopic Imaging

Each microchannel was coated with 10 μg/mL laminin (Life Technologies, 23017-015) for 1 hr at 37 °C prior to cell incubation. After aspirating excess unbound laminin, 0.1 μl of PPC and RPC suspensions (2 × 10^5^ cells/mL) in NB media supplemented with 10% FBS were then injected into the microchannel for a 2 hr incubation period. The SRC, SKC and bridged channel were also filled with media. NB media only (control) and NB media containing 100 ng/ml SDF are then added drop-wise into the SRC till the solution makes contact with media in the bridge channel. Cells were pre-incubated in AMD3100 for 30mins at 37 °C/5% CO_2_ for inhibition studies in microchannels. The bridged μ-Lane was then mounted on the motorized stage of an inverted microscope (Nikon Eclipse Ti) housed in a humidified incubator (Okolabs, NA, Italy). The temperature in the incubator was maintained at 37 °C with 5% CO_2_/balanced air supply. Live cell images were obtained at hourly intervals for 48 hrs in three to five independent experiments per experimental condition. Emitted fluorescence from GFP^+^-RPCs and PPCs was detected via a cooled CCD camera (Cool SNAP EZ, Photometrics, Tucson, AZ) and collated with Nikon software (NIS Element 4.3AR, Morrell Instrument Co. Inc., Melville, NY).

### Microfluidic Data Analysis

Cell motility parameters of maximum Euclidean and Accumulated distances and COM vector resolution in the Y-axis direction for each cell tracked over 48 hrs, were resolved via sequential use of Nd-to-Image6d (National Institutes of Health, Bethesda, USA), Manual Tracking (Fabrice Cordelières, Institut Curie, Orsay, France), and Chemotaxis and Migration Tool 2.0 (ibidi, Munich, Germany) plug-ins, all running on an ImageJ platform as previously described^97^. COM is a strong parameter for evaluating chemotaxis, and measures the spatial average displacement of all cell endpoints with positive or negative coordinates, depending on the direction of movement of a single cell or a population of cells. COM displacement in the Y-axis further specifies directionality of cell movement towards the SRR. Cell tracking data selected for analysis included only video recordings of RPC and PPC movement between 18 and 42 hrs to account for complete 24 hr periods of sustained steady-state gradients of SDF-1α within the microchannel. A two-sample Student’s t-test (t) assuming unequal variances was performed to assess significant difference in mean motility parameters between control and test groups.

## Additional Information

**How to cite this article**: Unachukwu, U. J. *et al*. Predicted molecular signaling guiding photoreceptor cell migration following transplantation into damaged retina. *Sci. Rep*. **6**, 22392; doi: 10.1038/srep22392 (2016).

## Supplementary Material

Supplementary Information

## Figures and Tables

**Table 1:**
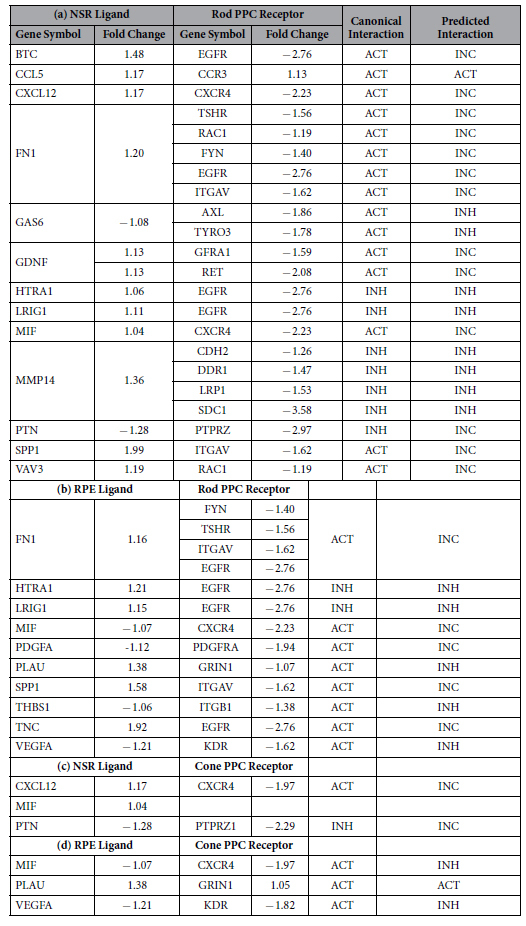
Top predicted ligand-receptor pairs guiding PPC migration.

To identify migratory ligand-receptor interactions, we correlated ligand and receptor expression states to downstream function in at least eighty-percent of the top ten p-value-sorted cellular movement subcategories. Activation or Inhibition are used to describe the effect of the ligand-receptor binding on downstream migratory signaling in both canonical and predicted (expression level based) categories. Significantly predicted ligand-receptor pairs include: a) NSR/Rod PPC with the chemoattractant CXCL12 and its CXCR4 receptor, fibronectin (FN1) and RAC1 interactions, b) RPE/Rod PPC with inflammatory cytokine SPP1-ITGAV (α-integrin), VEGFA-KDR, macrophage migration inhibitory factor (MIF) and CXCR4, c) NSR/Cone PPCs with CXCR4 receptor binding to both CXCL12 and MIF, both strongly predicted to affect cellular movement function determined by the gene expression states and d) RPE/Cone PPCs with macrophage migration inhibitory factor (MIF) and CXCR4.

**Table 2 t2:** Descriptive statistics for PPC and RPC migratory dynamics in microfluidic SDF-1α gradients.

Cell Type	Number of Tracked Cells(N)/([SDF] ng/ml)	Max. Accumulated Distance (Mean ± SEM)			Max. Euclidean Distance (Mean ± SEM)			Center of Mass – Y axis (Mean ± SD)		
		0	100	P-value	0	100	P-value	0	100	P-value
RPCs	438/(0) 242/(100)	403.37 ± 18.9	460.86 ± 28.2	0.0925	144.42 ± 8.97	176.98 ± 11.1	0.0242*	−6.64 ± 37.86	16.23 ± 47.26	0.0135*
PPCs	476/(0) 394/(100)	224.77 ± 18.2	87.27 ± 16.3	<0.0001*	179.36 ± 14.2	67.49 ± 15.2	<0.0001*	−31.75 ± 62.73	−1.78 ± 17.36	0.0033*

Data analyzed from live-cell imaging to generate Figure 6 include: number of tracked cells, maximum accumulated distance, maximum Euclidean distance and Center of Mass – Y-axis. For each parameter mean, standard error of mean (SEM) and p-values of independent samples t-tests are presented.
