# Supplementary material for: Predicted molecular signaling guiding photoreceptor cell migration following transplantation into damaged retina
Source: Sci Rep. 2016 Mar 3;6:22392. doi: 10.1038/srep22392 (PMC4776098; doi:10.1038/srep22392)
Supplement: Supplementary Information [file srep22392-s1.doc]

**Predicted molecular signaling guiding photoreceptor cell migration following transplantation into damaged retina**

Uchenna John Unachukwu^1,2^,, Alice Warren^2^, Ze Li^2^, Shawn Mishra^3^, Jing Zhou^2,4^, Moira Sauane^2^, Hyungsik Lim^5^, Maribel Vazquez^3^, and *Stephen Redenti^1,2^

^1^Biochemistry Doctoral Program, The Graduate School, City University of New York, New York, NY, USA

^2^Department of Biological Sciences, Lehman College, City University of New York, Bronx, NY, USA

^3^Department of Biomedical Engineering, City College of New York, City University of New York, NY, USA

^4^Neuroscience Doctoral Program, The Graduate School, City University of New York, New York, NY, USA

^5^Departments of Physics and Biology, Hunter College of the City University of New York, New York, NY USA

**Supplemental Material**

Supplemental Table 1


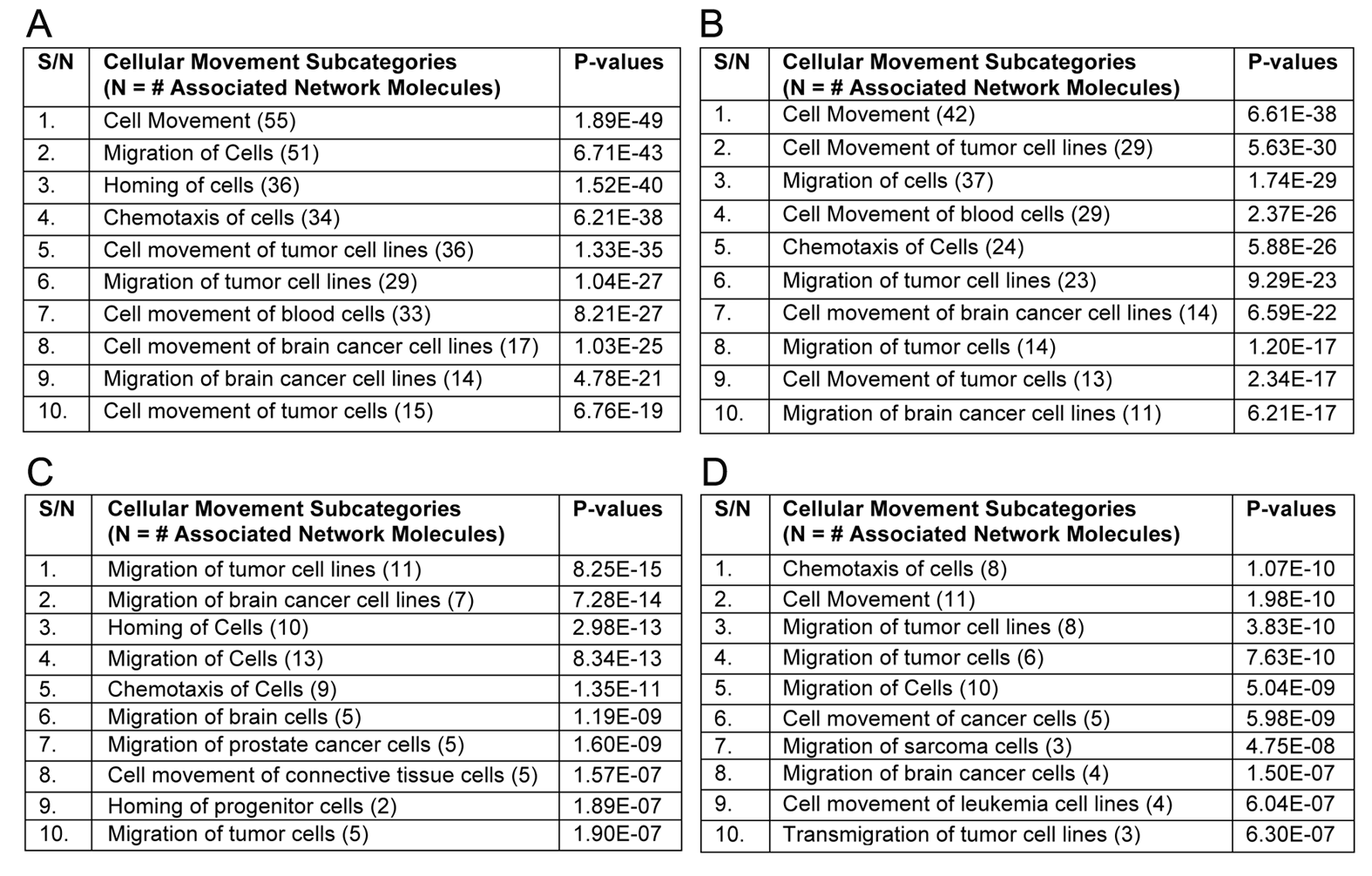


**Supplemental Table 1: Functional Categorization of Motility Deterministic Network Pathway Molecules**. Motility-deterministic networks of ligand-receptor interactions resolved from our matched datasets were assessed for involvement in the top ten cell movement functional sub-types curated in the IPA, based on their expression state using a right-tailed Fisher Exact test. A) Displays analysis results for the NSR/Rod network with the number of network genes involved in each cell movement category placed in parenthesis. P-values score the probability that network genes at their expression states activate or inhibit each respective cell movement subcategory, and are used in ranking the functional sub-types. B) RPE/Rods network genes were also categorized for cell motility function along with C) NSR/Cone and D) RPE/Cone networks. Genes with significant activating or inhibiting effects in at least 8 out of these top 10 cell movement subcategories are presented in Table 2 and represent our repertoire of candidate molecules for in vitro testing of their effects on cell motility and for designation of their downstream signaling interactions.


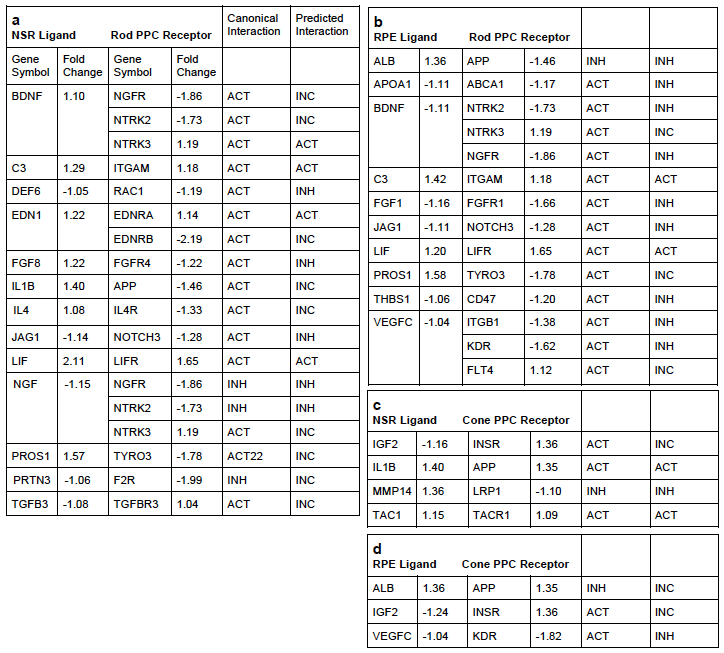


**Supplemental Table 2: Damaged Retinal ECM Ligands Matched to Cognate Cell Surface PPC Receptors and Predicted to Exhibit Downstream Signaling Associated with Migration**. Motogenic ligands from light-damaged retinal tissues interacting directly with PPC cell surface receptors and scoring lower than 80% activation of cell movement subcategories. Ligand-receptors pairs a matched for A) NSR/Rod, B) RPE/Rod, C) NSR/cone, and D) RPE/cone. The canonical interactions between the ligands and receptors are presented along with the predicted interactions between gene pairs based on their expression states. Predictions of receptor response to ligands include activation (ACT), inhibition (INH) or Inconsistent (INC) indicating that expression states differ from other cell type receptor gene expression in the IPA database used to predict downstream activation or inhibition of the receptor gene.
